# Supplementary figures and images for: Simultaneous targeting of XPO1 and BCL2 as an effective treatment strategy for double-hit lymphoma
Source: J Hematol Oncol. 2019 Nov 21;12:119. doi: 10.1186/s13045-019-0803-9 (PMC6868798; doi:10.1186/s13045-019-0803-9)

Figure S1

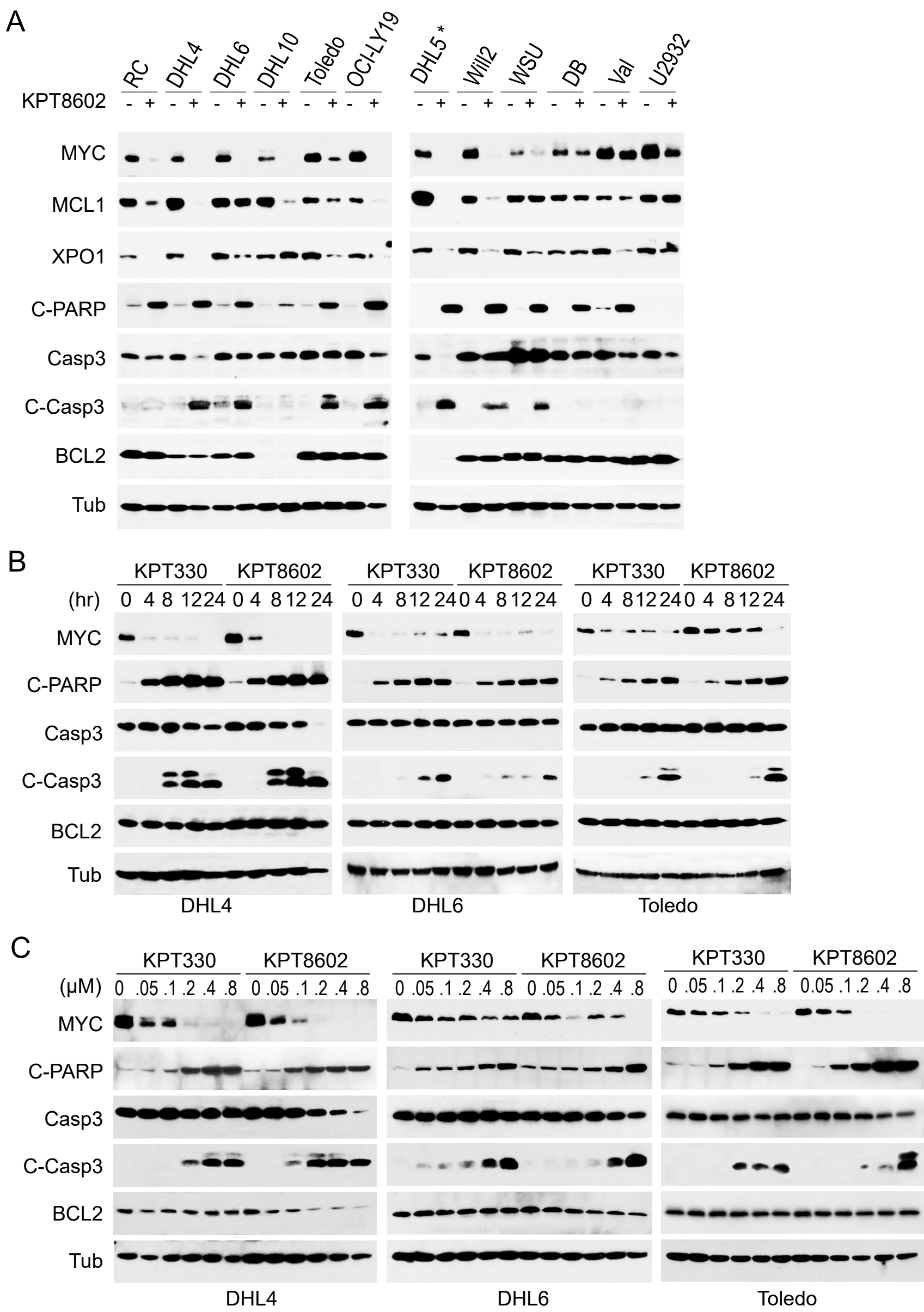

Figure S2

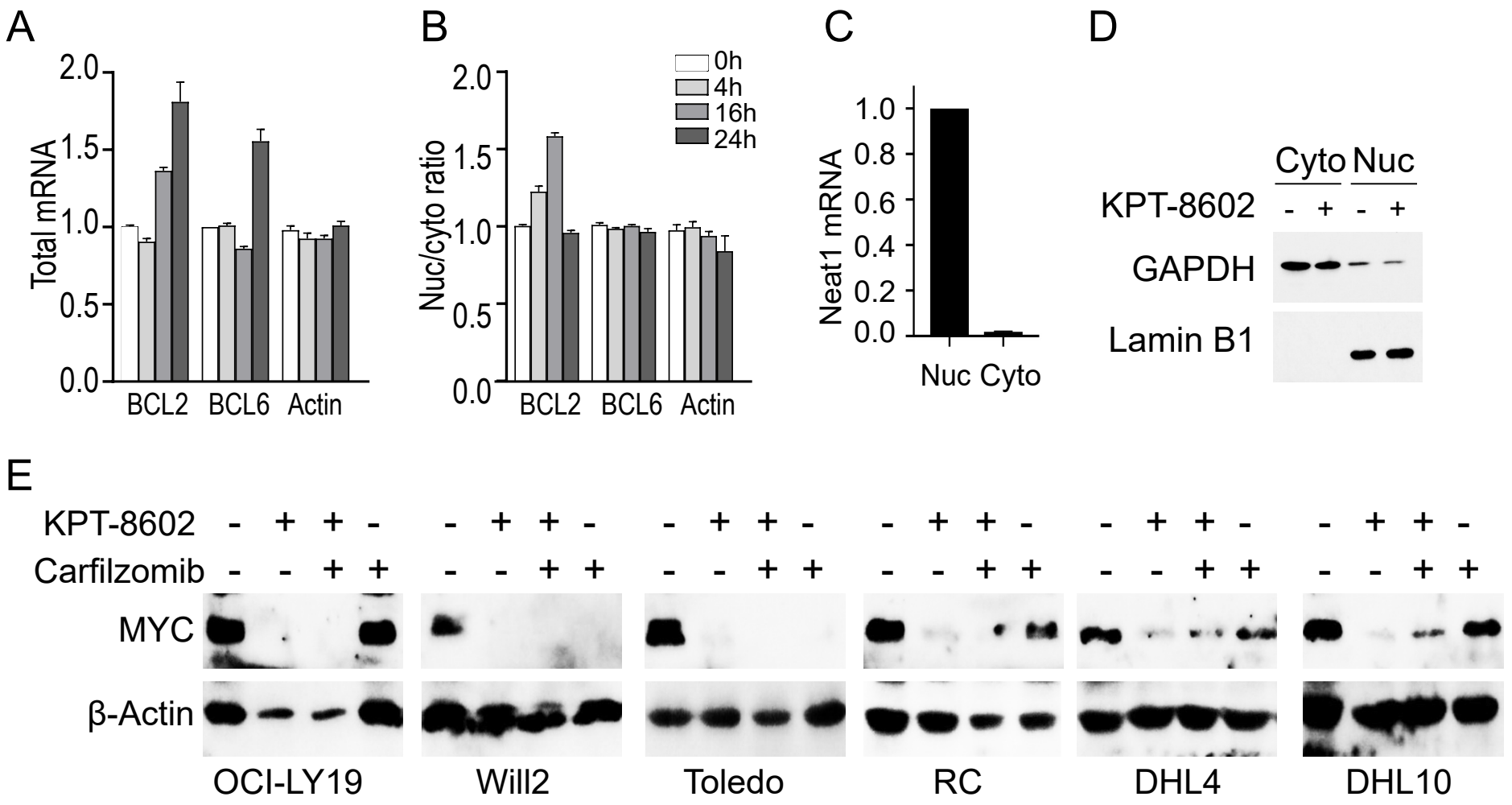

Figure S3

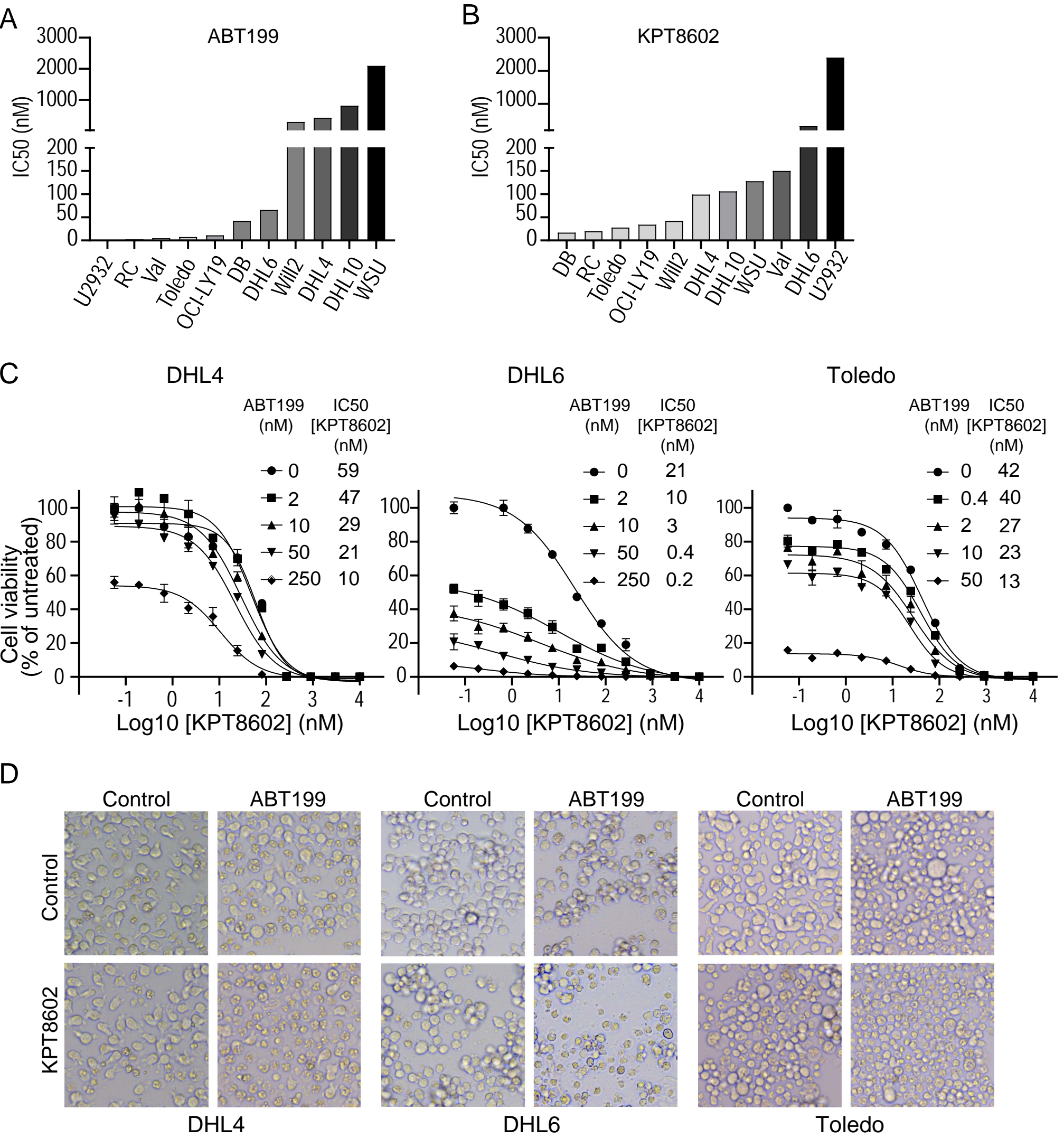

Figure S4

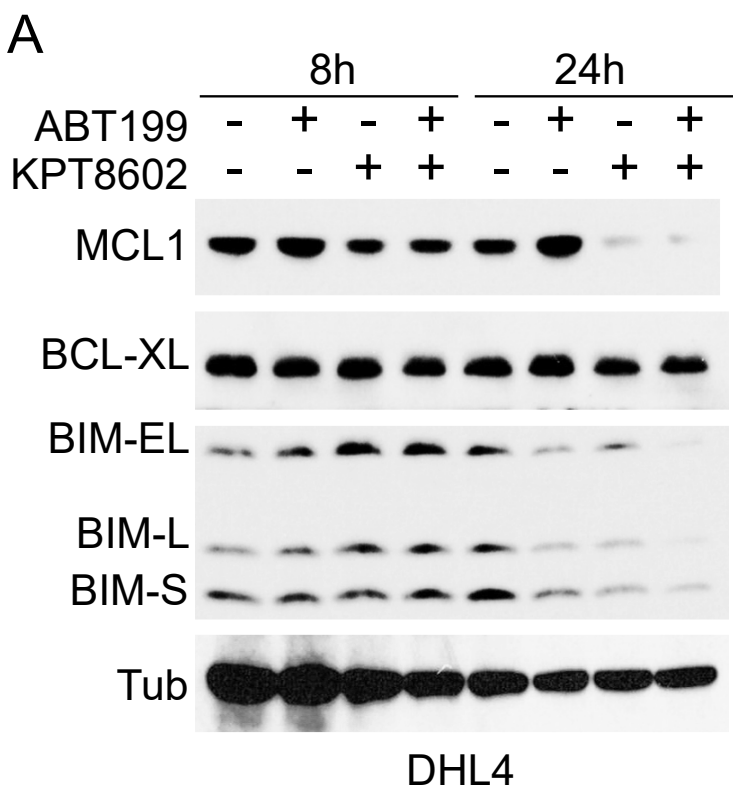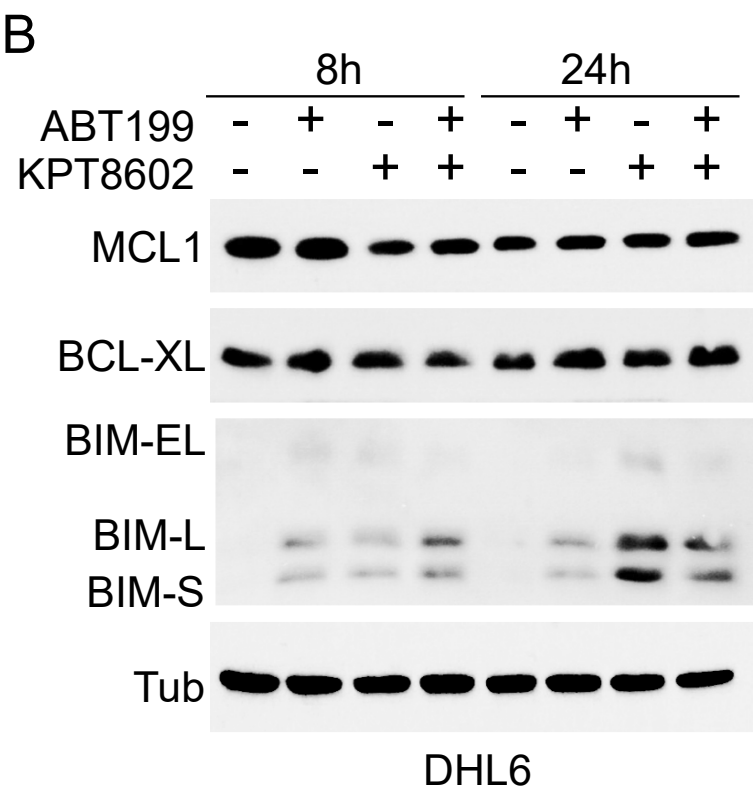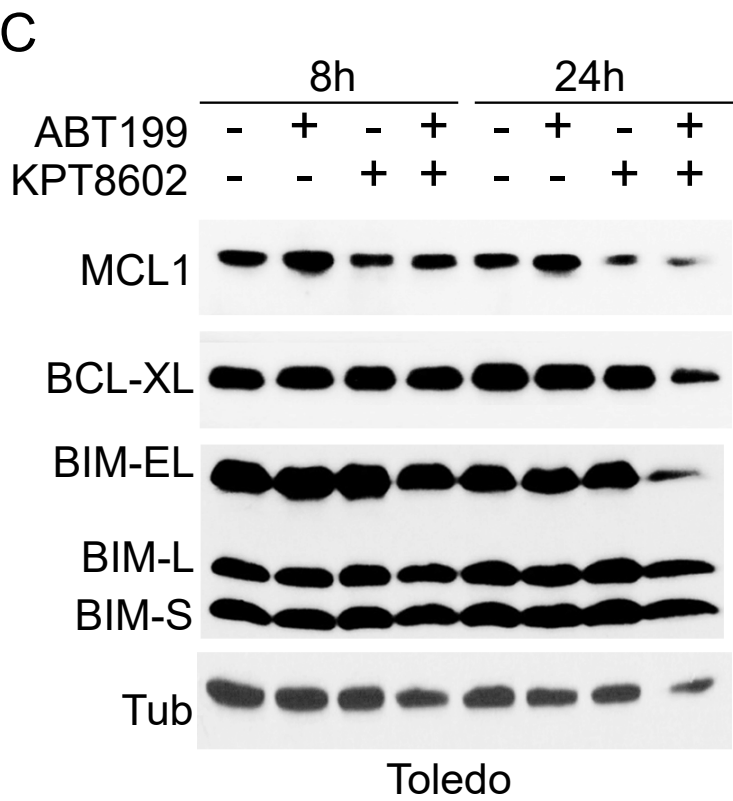

Figure S5

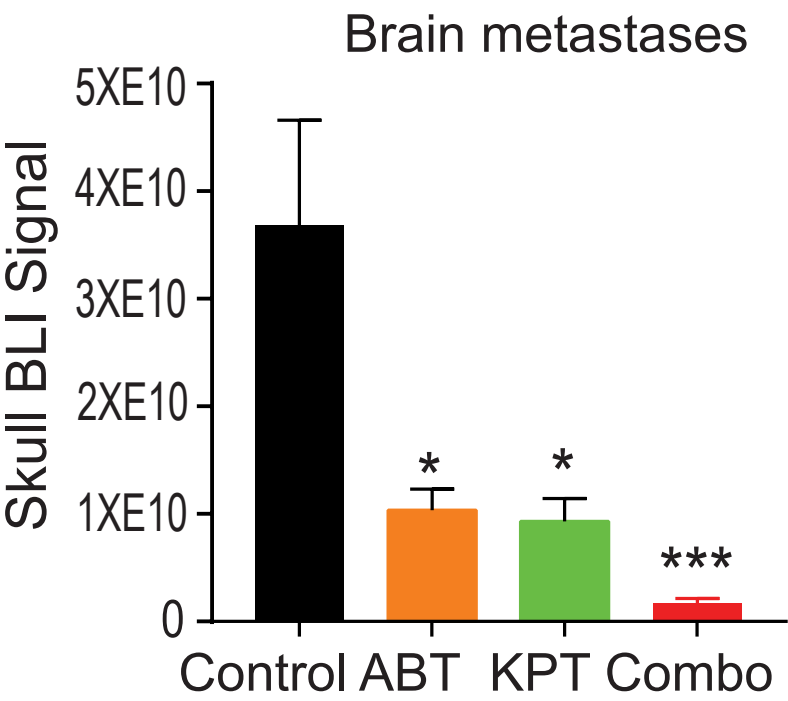

Supplement: Supplementary file 1 — Additional file 1: Fig S1. (A) Western blot analysis of MYC, MCL1, XPO1, and PARP/Caspase 3 cleavage. A panel of DHL cell lines was treated with 1 μM KPT8602 for 24 h. (B) Western blot analysis of MYC and PARP/Caspase 3 cleavage upon XPO1 inhibition in three DHL cell lines. (C) Western blot analysis of MYC and PARP/Caspase 3 cleavage upon treatment with different concentrations of XPO1 inhibitors for 24 hours. Fig S2. (A) Total mRNA and (B) nuclear to cytoplasmic ratios of BCL2, BCL6 and β-tubulin in DHL6 treated with 1 μM KPT8602. All mRNA levels were normalized to GAPDH. (C) Quantification of nuclear and cytoplasmic Neat1 mRNA levels by real-time PCR. (D) Analysis of nuclear and cytoplasmic GAPDH (cytoplasmic marker) and Lamin B1 (nuclear marker) by Western Blot. (E) Representative DHL cells were treated with 1 μM KPT8602 and/or 10 nM Carfilzomib for 24 hours. Fig S3. (A) IC50 values for ABT199 in a panel of DHL cell lines. (B) IC50 values for KPT8602 in a panel of DHL cell lines. (C) Cell viability in DHL cells treated with KPT8602 and ABT199 for 72 hours. The IC50 values for KPT8602 were calculated in the presence of different concentrations of co-administered ABT199. (D) Cell morphology of DHL cells treated with KPT8602 (100 nM) and ABT199 (40 nM for DHL4/DHL6, and 20 nM for Toledo) for 48 hours. Fig S4. Western blot analysis of MCL1, BCL-XL, and BIM proteins in DHL4 (A), DHL6 (B), and Toledo (C) cells. The drug treatment is the same as Fig 3b-d. Fig S5. Quantification of BLI signals from the crania of the tumor bearing animals following drug treatment. BLI signal data were presented as mean + standard error of mean. Two-tailed t test. * Control vs ABT199, P = 0.02; ** Control vs KPT8602, P = 0.01; *** Control vs Combination, P = 0.0008. [file 13045_2019_803_MOESM1_ESM.pdf]
